# Frequency Domain-optical Coherence Tomography of Coronary Arteries Using a Diluted Iodinated Contrast-saline Mix with 5-Fr Guide Catheters

**DOI:** 10.7759/cureus.4892

**Published:** 2019-06-12

**Authors:** Zoltan Varga, Naveen Rajpurohit, Shenjing Li, Tomasz Stys, Adam Stys

**Affiliations:** 1 Cardiology, University of South Dakota, Sanford Heart Hospital, Sioux Falls, USA; 2 Cardiology, Sanford Bemidji Heart and Vascular Center, Bemidji, USA

**Keywords:** optical coherence tomography, coronary artery disease, radial artery access

## Abstract

Background

Optical coherence tomography (OCT) is currently mostly performed using 6-Fr coronary guide catheters via femoral access. Catheters with such large internal diameters are necessary to deliver viscous contrast media and achieve sufficient red blood cell washout. Currently, undiluted iodinated contrast media (15 mL/injection) is used to clear the coronary arteries of red blood cells (RBCs). This leads to an increase in the total amount of contrast used and often the need for femoral artery access. Our objective is to assess the feasibility of performance of OCT using a 5-Fr guide catheter via radial access using diluted iodinated contrast.

Methods

We present a case series of 11 patients where second-generation frequency domain (FD)-OCT was used to assess the coronary arteries using a novel 70:30 dilution mixture of iodinated contrast medium with heparinized normal saline. All procedures were performed with a 5-Fr coronary guide catheter via the radial artery approach.

Results

All procedures were successfully performed vial radial access with good quality imaging obtained. The target vessel was the left anterior descending artery in eight patients, the right coronary artery in two patients, and the left main coronary artery in one patient. OCT resulted in a change in management in 7/11 (64%) patients; no complications were reported with OCT. On average, 10 mL of contrast was used per injection.

Conclusions

The current study demonstrates the feasibility of FD-OCT using 5-Fr guide catheters and diluted iodinated contrast media. This approach lowers contrast exposure and potentially decreases vascular complications without sacrificing image quality.

## Introduction

Intravascular optical coherence tomography (OCT) is a catheter-based invasive imaging system with numerous clinical applications in cardiology. It is a relatively new technique that uses near infrared (IR) light (1250-1350 nm) to generate cross-sectional images of the coronary arteries. Near-IR light has a shorter wavelength and higher frequency than ultrasound; thus, OCT images have 10-fold higher resolution, at the expense of reduced tissue penetration [1].

Clinical applications of OCT include diagnostic assessment of coronary atherosclerosis, risk stratification of thin-cap fibroatheromas, and studying lumen geometry to evaluate coronary disease severity and guide interventional procedures [2-3]. The modality of OCT is more sensitive than the intravascular ultrasound at detecting mechanical complications caused by stent placement, such as incomplete stent expansion, incomplete apposition, stent edge dissection and tissue protrusion, etc [4]. In bioreabsorbable stent implantation, OCT has many advantages over intravascular ultrasound because OCT is more sensitive to scaffold integrity, apposition to the coronary artery wall, and complications including dissection, thrombus formation, strut fracture, and changes in the struts over time. OCT has been recommended as standard imaging for bioreabsorbable stent implantation.

OCT requires clearance of red blood cells (RBCs) during image acquisition as RBCs attenuate near-IR signals. New frequency domain (FD)-OCT systems allow accelerated pullback speeds and permit the use of a single, high rate (4 mL/s) bolus injection of undiluted iodinated contrast to produce a blood-free environment [5]. Currently, OCT is performed using 6-Fr guide catheters usually via the femoral approach because a larger internal diameter is needed to deliver viscous contrast media and achieve good RBC washout without generating high pressure in the delivery system. Lately, experiments with other solutions used for RBC washout showed promising results [6]. In the last decade, we saw significant improvements in the design of coronary catheters and other interventional equipment, leading to an ongoing, gradual movement towards a radial approach in coronary interventions. With the increase in the number of coronary angiograms and interventions conducted through 5-Fr radial access, the need to perform OCT via 5-Fr radial access has also increased. Herein, we describe to our knowledge the first case series where OCT was performed successfully using a 5-Fr guide catheter via the radial artery with a 70:30 mix of iodinated contrast media diluted by heparinized normal saline to facilitate RBC washout without increasing the injection pressure in the 5-Fr catheter system.

## Materials and methods

Study population

From July 2011 to August 2013, we performed ad hoc coronary artery FD-OCT in 11 patients. Patients presented with stable angina or acute coronary syndrome and were found to have lesions of intermediate significance (50-70 %) on diagnostic coronary angiography. We excluded patients with New York Heart Association Class III or IV heart failure, unstable hemodynamic status, and/or renal dysfunction (serum creatinine >1.5 mg/dL).

Image acquisition protocol

Examinations were performed via radial approach using 5-Fr sheaths placed into the radial artery. All patients were given a heparin bolus (50-100 U/kg) to reach a target activated clotting time of more than 250 s. After placement of the 5-Fr guide catheter into the coronary ostium, a standard 0.014-in balanced middleweight (BMW) coronary guidewire (Abbott Medical, Santa Ana, CA, USA) was advanced into the coronary artery in a conventional manner. The 2.7-Fr C7 Dragonfly FD-OCT catheter (St. Jude Medical, St. Paul, MN, USA) was advanced over the 0.014-in BMW coronary guidewire through the 5 Fr guide catheter. The imaging lens of the catheter was positioned distal to the lesion of interest under fluoroscopic guidance.

In each patient, FD-OCT was performed with premixed, dilute, iodinated contrast medium to displace RBCs. This contrast medium consisted of 70 mL of Visipaque (GE Healthcare, Piscataway, NJ, USA) and 30 mL of heparinized normal saline solution. The contrast mix was prepared by discarding 30 mL of Visipaque 320 (used for flushing the intravascular OCT catheter prior to advancing over the BMW wire) from the standard 100 mL bottle and infusing 30 mL of heparinized normal saline solution back in. Thus, a 70:30 mix of iodinated contrast and heparinized normal saline was delivered as the flushing medium for image acquisition. An automated contrast delivery system (ACIST Medical Systems, Eden Prairie, MN) was used for contrast delivery through the guide catheter at a rate of 4 mL/s, zero rate of rise and pressure of 300 psi. An average of 14 mL of dilute contrast was used in each run. We acquired data using a commercially available FD-OCT system (C7-XR, OCT Imaging System, St. Jude Medical). Images were calibrated by automated adjustment of the Z-offset, and an automated pullback was set at 20 mm/s for 2.5 s. Hemodynamic and electrocardiogram changes were recorded during each injection of contrast for image acquisition.

Image analysis

We performed FD-OCT analyses using the dedicated software with an automated contour detection algorithm (Offline Review Software version C.0.2, St. Jude Medical). All frames from FD-OCT (C7) were analyzed by two interventional cardiologists. The observers inspected each OCT pullback on a frame-by-frame basis along the length of the pullback images. The OCT procedure was considered successful if the target lesion was adequately imaged, as per usual practice.

## Results

Baseline characteristics are shown in Table 1. 

**Table 1:** Baseline characteristics LAD: left anterior descending artery; RCA: right coronary artery; LMCA: left main coronary artery; CAD: coronary artery disease

	Patients (n=11)
Men	6
Age (years)	57-85
Indication of stable angina	8
Indication of acute coronary syndrome	3
LAD as target vessel	8
RCA as target vessel	2
LMCA as target vessel	1
Hypertension	9
Diabetes Mellitus	6
Prior CAD	9
Dyslipidemia	7
Smoking	5

**Figure 1:**
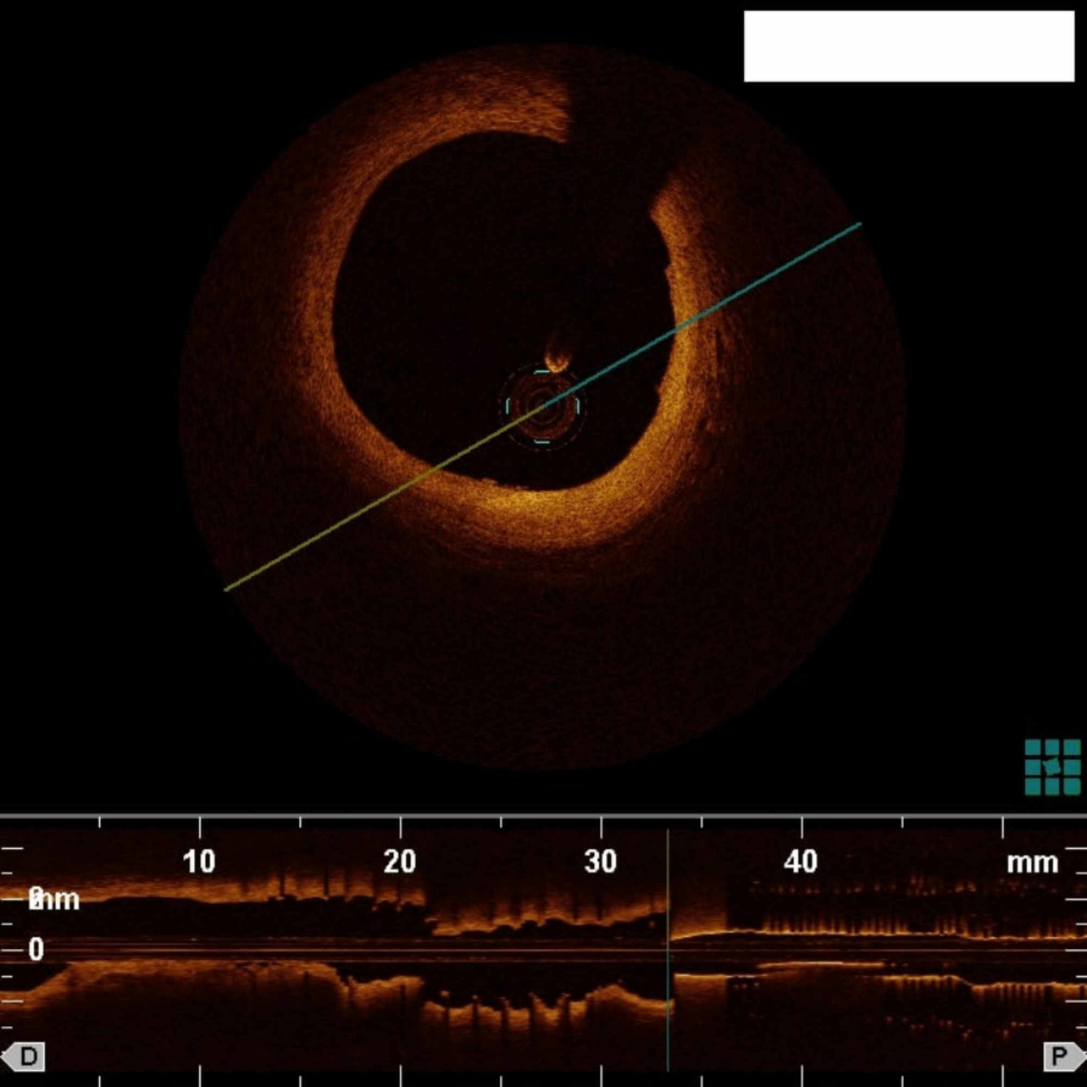
OCT of the left main coronary artery using diluted iodinated contrast medium and a 5-Fr guide catheter OCT:Optical coherence tomography

Six out of the 11 patients were men, age ranged from 57 to 85 years. No change in the access site to femoral was necessary for any of the patients. The majority of patients had stable angina (8/11, 73 %) with preserved left ventricular ejection fraction. In three patients, OCT was performed for assessing lesions in the setting of acute coronary syndromes. Target vessels included the left anterior descending artery in eight patients, right coronary artery in two patients, and the left main artery in one patient (Figure 1). 

**Figure 2:**
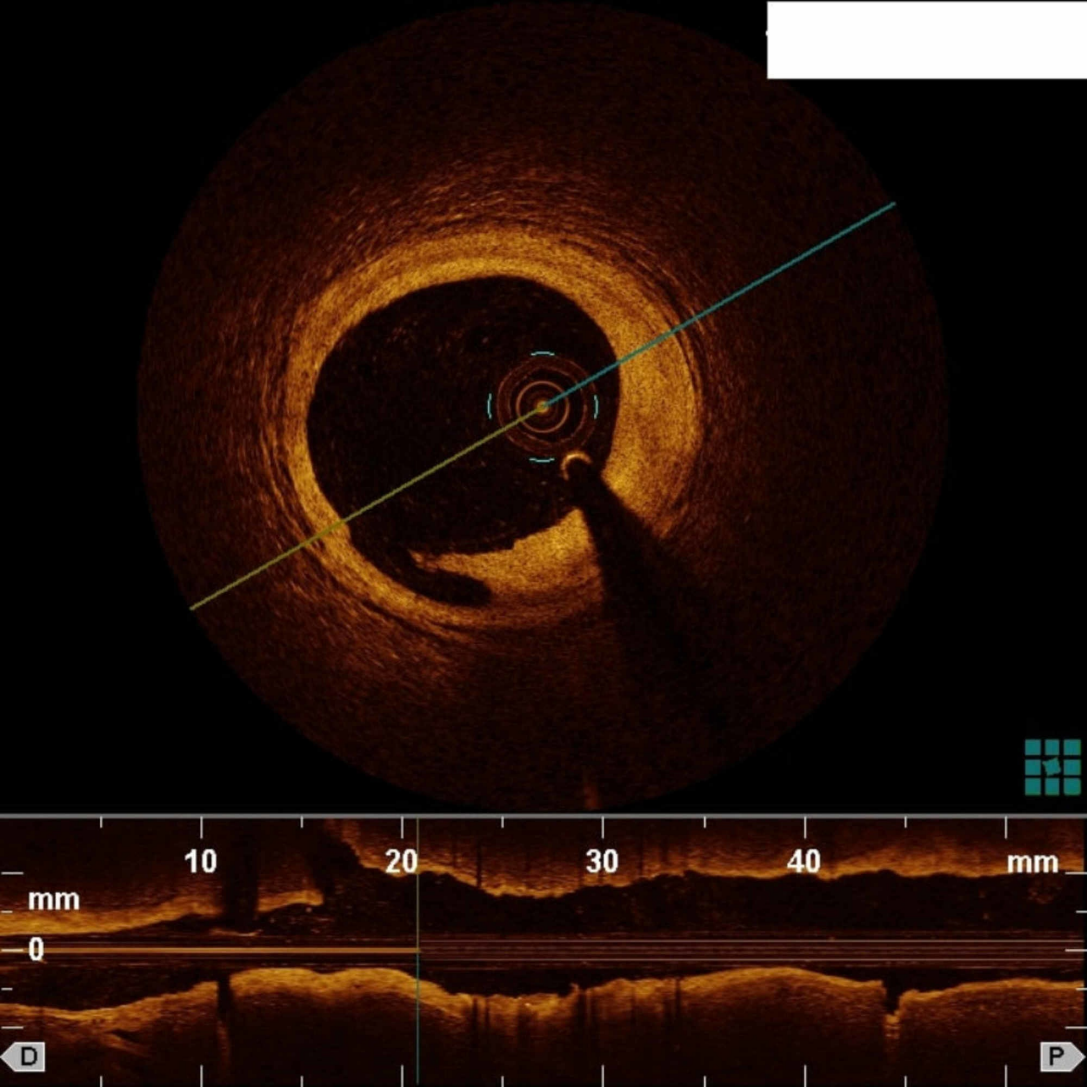
OCT using diluted iodinated contrast medium and a 5-Fr guide catheter showing a dissection at the 7-o’clock position OCT: Optical coherence tomography

**Figure 3:**
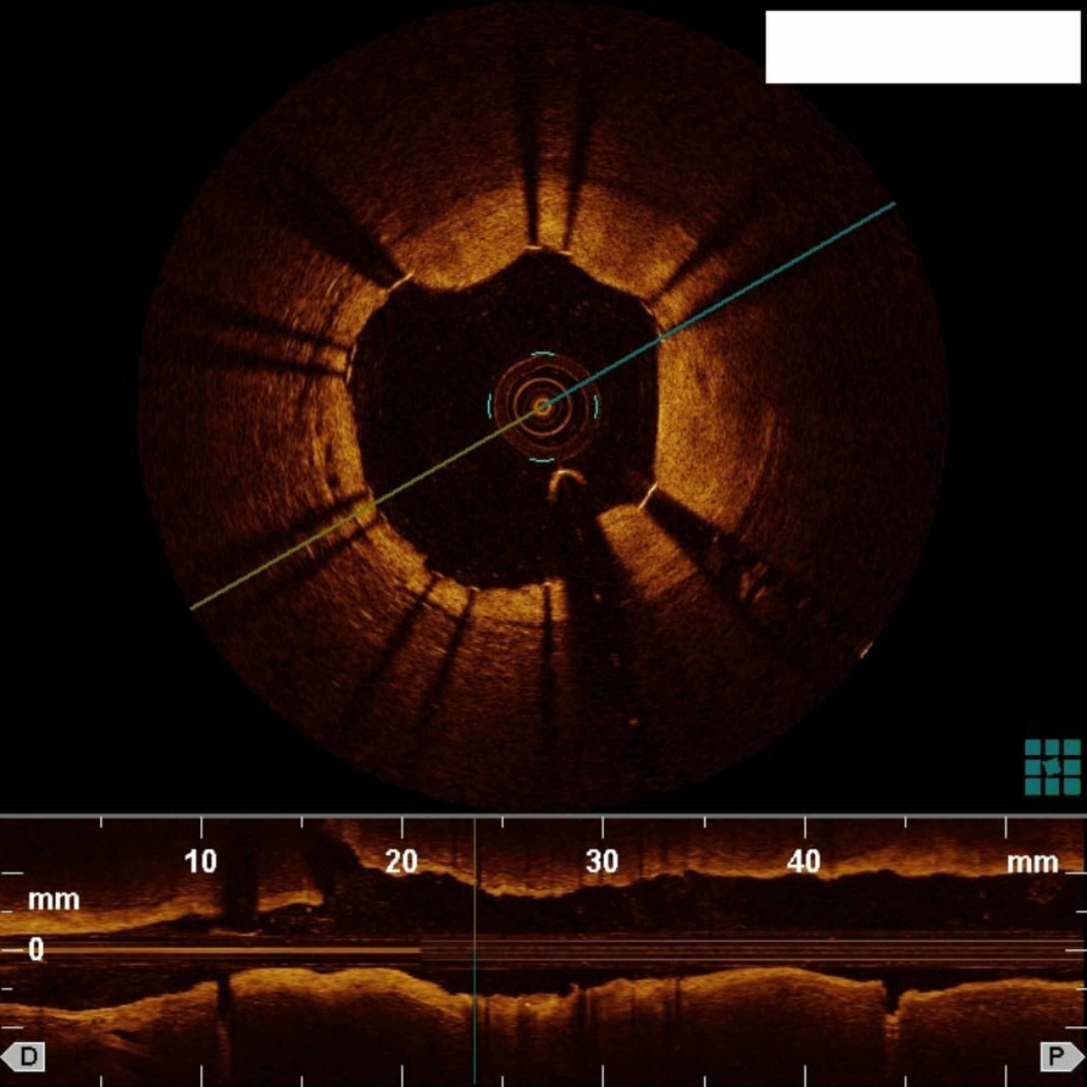
OCT using diluted iodinated contrast medium and a 5-Fr guide catheter showing adequate stent apposition OCT: Optical coherence tomography

We successfully evaluated all lesions via OCT with subsequent image-guided intervention in 7/11 (64 %) patients. No complications were reported during or immediately after OCT. OCT images of a coronary artery dissection and a well placed coronary stent are shown in Figure 2 and Figure 3, respectively.

## Discussion

FD-OCT overcomes many of the technical limitations of conventional time domain-OCT. It eliminates the need for balloon occlusion of the coronary arteries reducing vessel injury and potential ischemia, and enables faster image acquisition (100 frames/s) with pullback speeds up to 20 mm/s with the potential to scan 4-6 cm of the epicardial coronary artery in <5 s [1,7]. However, this requires a high-rate bolus injection of iodinated contrast media (15 mL) to produce a blood-free environment.

The current study was performed to evaluate the use of FD-OCT in assessing coronary artery stenosis using a 5-Fr guide catheters via the radial approach. This requires dilution of the iodinated contrast medium because the use of undiluted contrast leads to the generation of high pressures in a 5-Fr system during flushing. We performed a serial dilution of the iodinated contrast media with heparinized normal saline in different ratios and acquired images with each. Excellent images were acquired using the 70:30 ratio (70 mL iodinated contrast media with 30 mL heparinized normal saline) without generating high pressures in the catheter system. OCT was successfully performed using a 5-Fr guide catheters through the radial artery in all cases.

Transradial coronary angiograms and interventions have revolutionized the world of cardiac interventions. Transradial coronary angiograms offer lower rates of periprocedural vascular complications, lower bleeding rates, and earlier patient ambulation compared to transfemoral routes [8]. Thus, the number of transradial interventions is on the rise in the USA [8-9]. Downsizing of devices and sheath sizes reduce procedural complications during transradial catheterization [10]. Because of the aforementioned reasons, a 5-Fr radial access is the current standard of practice at our institution for diagnostic coronary angiograms and for most percutaneous coronary interventions. Performing OCT during a transradial angiogram with a 5-Fr right radial sheath and guide catheter would require changing access to the femoral artery and/or upgrading to 6-Fr radial access. This could lead to an additional step, with potentially more access site complications, patient discomfort, longer procedure time, and need for additional equipment.

Herein, we also report OCT imaging of the left main coronary artery (LMCA). Conducting OCT through the radial artery is more challenging than through the femoral artery as guide catheter engagement in the ostium of the LMCA is technically more difficult. Guide catheter engagement is a crucial step in OCT because poor guide catheter engagement can lead to poor image acquisition as blood flow from the side of the catheter at the ostium of the LMCA can lead to poor RBC clearance and blood swirling artifact in the lumen of the artery. However, we were able to successfully image the LMCA with good acquisition quality (Figure 1).

Iodinated contrast is preferred over saline or Ringers’ lactate for flushing RBCs, because high viscosity solutions more completely remove RBCs [1]. Unfortunately, this can add to the total amount of contrast used and expose the patient to a higher risk of contrast-induced nephropathy. In general, it is important to reduce the amount of contrast media exposure to as low as possible. We used a 70:30 mix of iodinated contrast media diluted with heparinized normal saline to clear the vessel of RBCs during image acquisition. This strategy reduced the absolute amount of iodinated contrast media to 10 mL/injection, versus 15 mL used in conventional protocols, without compromising image quality and acquisition. By successfully reducing the absolute amount of contrast used per patient, we can potentially use more FD-OCT to guide percutaneous coronary intervention of patients with renal insufficiency.

Although OCT through the radial artery has been reported previously, most studies used catheters and sheaths with 6-Fr or larger internal diameters. To our knowledge, this is the first reported study wherein OCT was performed through the right radial artery using 5-Fr sheaths and guide catheters [11].

Limitations

Our study includes a small series of patients from a single institution. Lack of a control group is another limitation when evaluating images acquired using diluted iodinated saline contrast. Larger studies with a control group are needed to assess the safety and feasibility of this technique.

## Conclusions

The present study demonstrates the feasibility of FD-OCT via the radial approach using 5-Fr guide catheters. This technique eliminates the need to switch to femoral access and 6-Fr catheters if the case began with a 5-Fr radial. Furthermore, the use of diluted iodinated contrast decreases the patient's dye load without sacrificing image quality, and potentially can decrease the risk of contrast-induced nephropathy.
